# Effect of Ultraviolet C Disinfection Treatment on the Nanomechanical and Topographic Properties of N95 Respirator Filtration Microfibers

**DOI:** 10.1557/adv.2020.347

**Published:** 2020-12-01

**Authors:** Meng Yujie, Zeng Rae, Rubin Kurt, Barry Kelly

**Affiliations:** 13 KLA Corporation, 205 Perimeter Park, Suite C, Knoxville, TN 37922 USA; 23 1 Technology Drive, Milpitas, CA 95035 USA

## Abstract

Ultraviolet germicidal irradiation (UVGI) N95 filtering facepiece respirator (FFR) treatment is considered an effective decontamination approach to address the supply shortage of N95 FFRs during the ongoing Covid-19 pandemic. In this study, we investigated the nanomechanical and topographic properties of filtration fibers that have been exposed to different doses of UVC radiation. UVC exposure was shown to decrease both Young’s modulus (E), hardness (H) and fiber width, as measured on individual polypropylene (PP) fibers. Our results also show that the PP microfiber layer loses its strength when N95 respirators are exposed to an accumulated UVC dose during the process of decontamination, and the PP fiber width also exhibits a logarithmic decrease during UVC exposure. The nanoscale measurement results on individual fibers suggest that maximum cycles of UVC disinfection treatment should be limited due to excessive accumulated dose, which has the potential to decrease the fiber breaking strength.

## INTRODUCTION

The unprecedented Covid-19 epidemic has led to a critical shortage of personal protective equipment (PPE). N95 masks are one of the key PPEs to protect frontline healthcare workers, and these devices are intended to be used once and then disposed. The N95 filtering facepiece respirator (FFR) face mask is typically composed of multiple filtration layers, including those shown in Figure 1a, with the inner filtration layer considered to be the key functional component [1]. This layer is made from meltblown non-woven fabric, which is composed of thousands of very thin, noncontinuous fibrils. An electric charge on the fibers enables a 10 times higher filtration efficiency without adversely increasing the air resistance, due to a technique called corona electrostatic charging applied to the N95 filtration layer during manufacturing [2]. Polypropylene (PP) is the most widely used raw material for the meltblown fibrils due to its low melting point. Figure 1b shows a SEM image of the primary filtration layers.

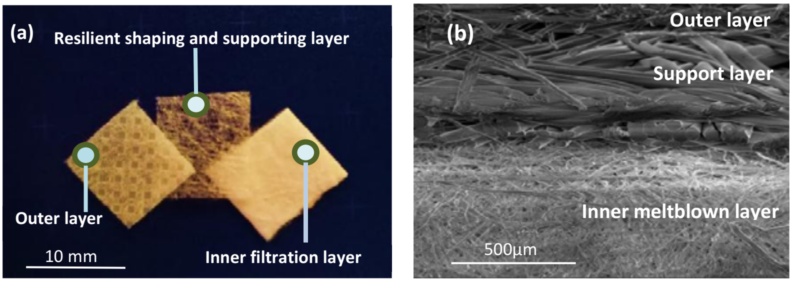

Figure 1. (a) An N95 respirator coupon sample shows the three layers of nonwoven material: the inner filtration layer is meltblown (non-woven) polypropylene to provide effective filtration; the middle layer provides the mask shape and supports the inner and outer layers; and the outer layer provides filtration for larger particles. The FFR model selected for this research was the 3M 9211+ N95 respirator, with the inner filtration layer selected for testing. (b) Scanning electron microscopy (SEM) image of the cross section of commercial N95 mask material, showing the electrocharged layers.

Due to the limited supply of N95 FFRs, the Centers for Disease Control (CDC) made recommendations on risk management approaches for the use of these masks and provided guidance regarding limited or extended reuse of N95 FFRs by healthcare workers. Based on the limited research available, three methods have shown the most promise in decontaminating N95 FFRs for reuse: (1) ultraviolet germicidal irradiation (UVGI), (2) vaporous hydrogen peroxide, and (3) moist heat [3]. Previous research demonstrated that the UVGI technique, which uses a wavelength range of light that falls in the ultraviolet C spectrum (short-wavelength ultraviolet, where $\lambda$ = 100-280nm), could inactivate influenza and coronaviruses in N95 FFRs by penetrating the mask and damaging the molecular bonds that hold together the nucleic acids of viruses and bacteria to prevent them from infecting and/or replicating in human cells [4]. The genomic materials DNA and RNA have a maximum absorption wavelength around 260nm [5,6]. As of August 2020, the UVC inactivation of the Covid-19 virus in N95 FFRs has not been demonstrated in peer-reviewed studies. However, UVGI decontamination has been widely applied in hospitals to inactivate airborne pathogens. Its efficiency is critically dependent on both UV wavelength (nm) and UVC dose (J/cm$^2$). When the UVC light passes through the multiple mask layers, the structure of the N95 FFR causes a drop in transmitted light intensity. Therefore, for effective decontamination, the required UVC dose at the N95 respirator surface is several hundred-fold greater than the dose needed for virus inactivation on surfaces or in the air. The currently suggested procedure for UVC treatment considers $1J/cm^2$ as the minimum acceptable UVC dose for N95 FFR decontamination [7].

There is risk of damage to the N95 respirator materials after multiple decontamination cycles, and one reason is that UV radiation degrades polymers. Lindsley et al. studied the effects of four different UVGI treatments on N95 respirator filtration performance and structural integrity, with a specific interest in how large a cumulative UVGI dose respirators can withstand [8]. Bursting strength tests on N95 structures composed of tangled and spun-bonded polypropylene fibers demonstrated that UVC treatment dramatically decreased the strength of N95 layers. There are also concerns about electrical charge loss by the filtration fibers during UVC treatment [9]. Considering these factors, it is important to understand the UVC effect on the nanoscale mechanical properties of the individual fibers.

## EXPERIMENTAL METHOD

A 3M 9211+ Aura Particulate Disposable Respirator Mask was cut into 1cm square coupons and placed into a Thermo Scientific Prisma scanning electron microscope (SEM). The SEM was operated at a low voltage of 1.00kV, and the respirator structure was studied using an Everhart-Thornley SE detector. The resulting SEM images are shown in Figures 2. Six additional coupons of the meltblown layer were cut from the same respirator and placed in a cardboard chamber and exposed to UVC light with a primary wavelength of 254nm. The chamber was equipped with two 38W parallel lamps, and the fiber samples were positioned 30cm from the lamps when exposed to the UVC light. Samples were left in the chamber for 5, 35, 65, 95 and 155 minutes, resulting in cumulative exposures of 1, 7, 13, 19 and $31J/cm^2$ of UVC, for a total of five test conditions, plus an untreated sample. The chamber temperature was monitored, with a mean temperature during exposure of 24.5°C. The nanoindentation measurements were conducted at 22°C and data were randomly collected on different positions of fiber and among different fibers A minimum of 10 tests were performed under each condition for statistical analysis. The UVC dose was defined as:

$$\text{UVC dose} \propto \frac{\text{UV bulb power} * \text{Exposure time}}{4 * \pi * \text{UV bulb distance}^2} \#(1)$$

where the UV bulb distance is 30cm, the distance between the lamps and the sample puck position. For nanoindentation measurement, the N95 respirator meltblown layer microfibers were prepared under the following protocol: (1) the PP microfibers were untangled and placed on painter's tape, pushed down firmly to make good adhesive contact with the tape; (2) a thin layer of epoxy was applied to the aluminum sample stub; (3) the microfibers were placed on the epoxy and pushed firmly to make solid contact with the aluminum stub; and (4) the tape was removed after the epoxy cured. The undersides of the microfibers were surrounded by epoxy resin, and the epoxy-free portions of the fibers were scanned using the KLA Nano Indenter G200X NanoVision option to characterize individual fibers. The nanoindentation testing was conducted using the Continuous Stiffness Measurement (CSM) option and an InForce 50 actuator fitted with a Berkovich indenter. With the CSM option, Young's modulus and hardness were measured as a continuous function of penetration [10]. The InView test method called

Advanced Dynamic E and H allowed us to measure Young's modulus and hardness of the PP microfibers.

The topography and width measurements of the fibers were obtained by imaging coupons with a KLA Zeta-20 optical profiler. The optical profiler enabled us to quickly obtain images and measurements of the PP fibers without contacting the sample, while eliminating potential SEM-induced influences on the fiber properties. For this study, the ZDot structured illumination imaging mode was used to produce 3D True Color images for measuring the PP fiber width. The fiber width sampling at each dose was based on five different sites measuring five fibers per site, for a total of 25 width measurements per dose.

## RESULTS AND DISCUSSION

Figure 1b shows the scanning electron microscopy (SEM) image for the layer cross section, taken from the 3M 9211+ N95 respirator. The inner filtration layer was made up of non-woven polypropylene fibers that were tangled to form a three-dimensional porous structure. Figures 2 shows the inner meltblown filtration layer microstructure at higher magnification. The average microfiber diameter was about 1-2μm.

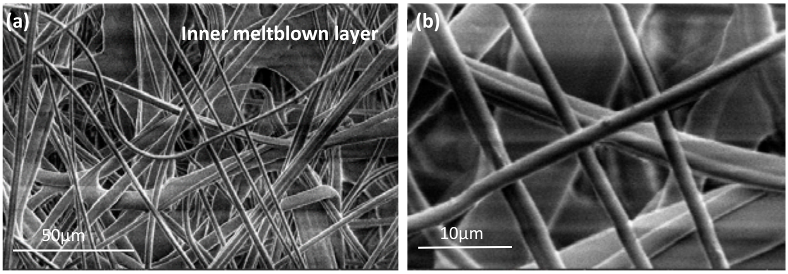

Figure 2. SEM image of (a) the inner filtration layer reveals the meltblown microfibers forming the porous network structure and (b) the individual meltblown microfibers.

Figure 3a shows a 16μm x 16μm scanning probe microscopy (SPM) image of an untreated meltblown microfiber, prior to nanoindentation, using NanoVision scanning on the KLA Nano Indenter G200X. Three nanoindentation tests were then performed on the fiber under maximum loads of 0.1mN, 0.3mN and 0.5mN respectively. Figure 3b shows the SPM image of the fiber post-indentation, where the three residual indentation marks are indicated. The load-displacement curves for the three tests are shown in Figure 3c, with the line color for each curve corresponding to the mark indicators. Indentation elastic recovery was observed on the unloading segments, generating different residual displacements. Using the 3D SPM image, the residual indentation size and depth were also measured, based on the surface profile along the longitudinal axis of the fiber. Residual depth measurements are shown in Figure 3d, where the loads of 0.1mN, 0.3mN and 0.5mN resulted in residual indentation depths of 57nm, 107nm and 147nm, respectively, which is consistent with the indentation load and depth curves. The inset image in Figure 5d shows the profile location on the fiber. For indentation measurements, loading was terminated at a penetration depth ≥ 500nm. The Young's modulus and hardness were determined on PP fibers that were exposed to different cumulative doses of UVC light. Young's modulus, E, is a measure of material stiffness, and is defined as:

$$E = \sigma/\epsilon \#(2)$$

where $\sigma$ is the uniaxial stress and $\varepsilon$ is the resulting strain, or proportional deformation of the material. Hardness, H, is a function of the maximum applied load and the resulting plastic deformation of the material, and is defined as:

$$H = P_{max}/A \#(3)$$

where $P_{max}$ is the maximum applied load and A is the area of the plastic deformation. The process of extracting E and H from equation (2) and (3) assumes that a Berkovich indenter drives into semi-infinite solid. However, indentation measurement is performed on a curved surface of PP fibers in this case. It is possible that Oliver-Pharr method results in underestimation of hardness because of the effects of free surface. Nevertheless, we adopted the same method for comparative purposes. The absolute value of PP fiber's nanomechanical properties will be impacted by the free surfaces, but its relative changes are the same.

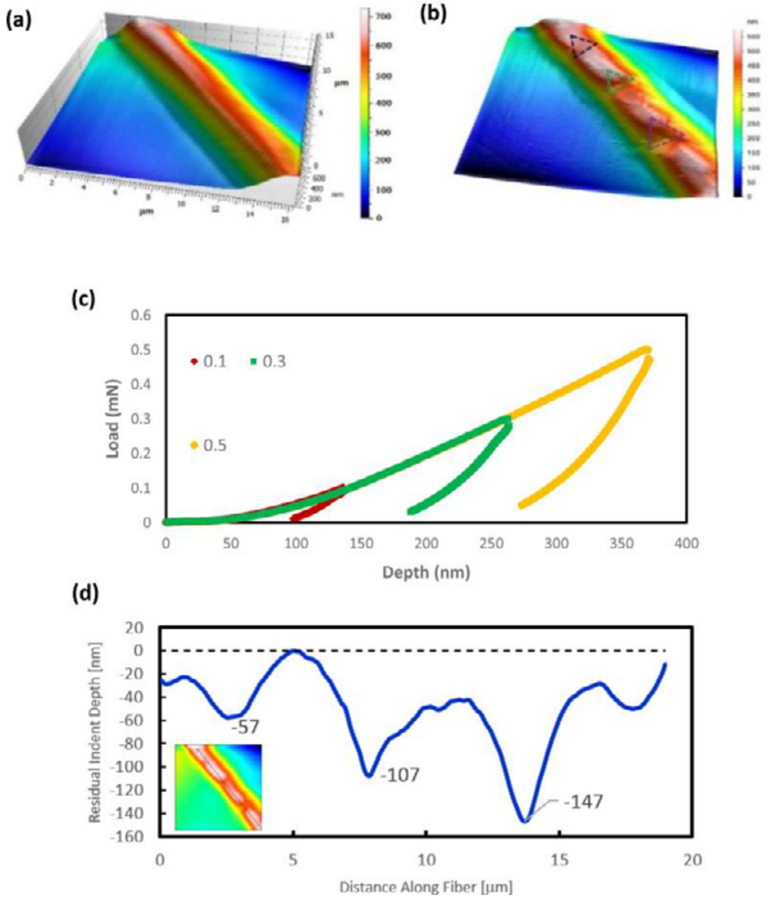

Figure 3. N95 respirator filtration fiber testing using the KLA Nano Indenter G200X with NanoVision scanning, showing an individual untreated meltblown non-woven fiber before (a) and after (b) nanoindentation, where the nanoindentation image highlights the three residual marks on the fiber. Scan field of view is 16µm x 16µm with peak to valley height of 0.6µm. Load-displacement curves corresponding to the three indentation tests are also shown (c), and a surface profile across the indentation marks measured the depth of each residual indentation (d), with the profile location on the fiber also shown (d, inset).

Figure 4 shows nanoindentation load and displacement curves of PP fibers for each of the five UVC doses. A noticeable difference between the indentation responses of the five samples is the displacement during the loading and holding segments. In general, when the UVC exposure dose was high, the curves shifted slightly to the right while the maximum load decreased significantly at the same indentation depth. The physical changes to the fibers at higher doses are reflected in the hardness and Young's modulus results. Figure 5 represents the indentation hardness (a) and Young's modulus (b) as a continuous function of depth for the five doses. Figure 6 extracts the PP fiber hardness (a) and Young's modulus (b) at an indentation depth of 500nm, where the quantities have largely stabilized, for the five different UVC doses. When the UVC dosage was raised from 0 to 1J/ $cm^2$, a precipitous drop in hardness and a significant drop in modulus were observed. Further increase of UVC dosage to 7J/$cm^2$ appears to result in a small increase of fiber hardness and possibly a small increase in modulus as well. At higher UVC doses, both hardness and modulus decrease monotonically with increased UVC dose. Overall, the observed relationship between UVC dose and reductions in hardness and modulus of the N95 fibers are consistent with previous research on the UVC sensitivity of polypropylene fibers [11]. Polypropylene has been reported to be especially susceptible to UV damage. It experiences photooxidative degradation in the presence of UV light due to breakage of the main molecular chains of the PP, causing brittleness and loss of tensile, impact and elongation strengths. Whether the lower dose regime of 1 J/$cm^2$ to 7 J/$cm^2$ represents a deviation from the overall monotonic relationship between dose and hardness/modulus would require further study.

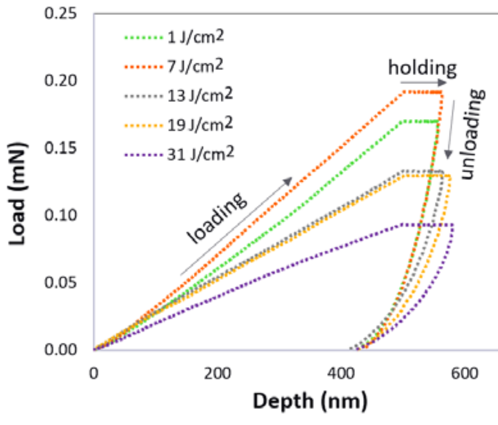

Figure 4. Nanoindentation load and displacement curves of PP fibers subjected to five different doses of UVC light. The maximum load decreases at higher doses. The arrows indicate the depth response versus load in the order in which the loading was applied.

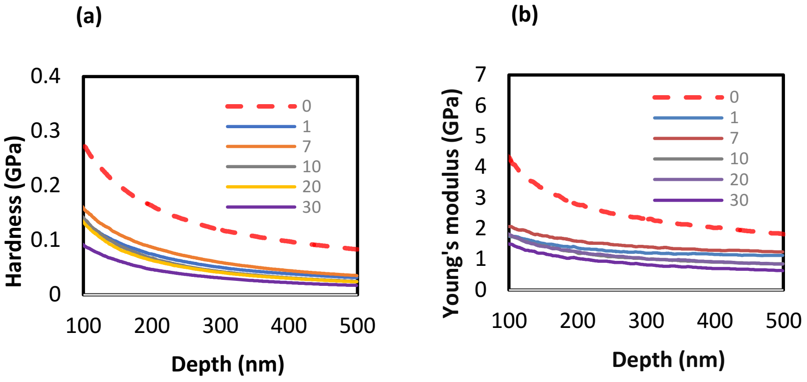

Figure 5. Nanoindentation hardness (a) and Young's modulus (b) of PP fibers subjected to five different does of UVC light. The untreated fiber measurements are represented by the dotted lines.

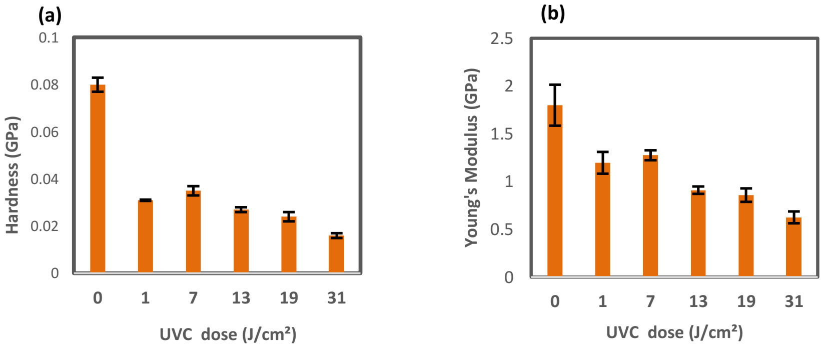

Figure 6. The hardness (a) and Young's modulus (b) of PP fibers exposed at different UVC doses. Data are reported for an indentation depth of 500nm.

Figure 7 shows a Zeta-20 image of the N95 respirator meltblown layer with no UVC treatment. The untreated fibers appear to be tangled and variable in size and shape, with some fibers joining to form large bundles.

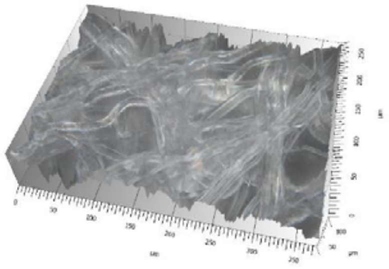

Figure 7. Zeta-20 image of the untreated N95 respirator meltblown layer. Gray level variation is due to translucence of the sample fibers and the dark sample mounting surface.

Figure 8 shows the N95 respirator meltblown layer topography following different UVC treatment conditions, with height variation represented by color. With no UVC exposure, the fibers appear wider and more bundled. Voids between the fibers appear smaller than the UVC-treated samples. Following UVC treatment, the fiber width shrinks for doses greater than 1J/cm$^2$. At higher doses, the fibers also appear to separate from each other.

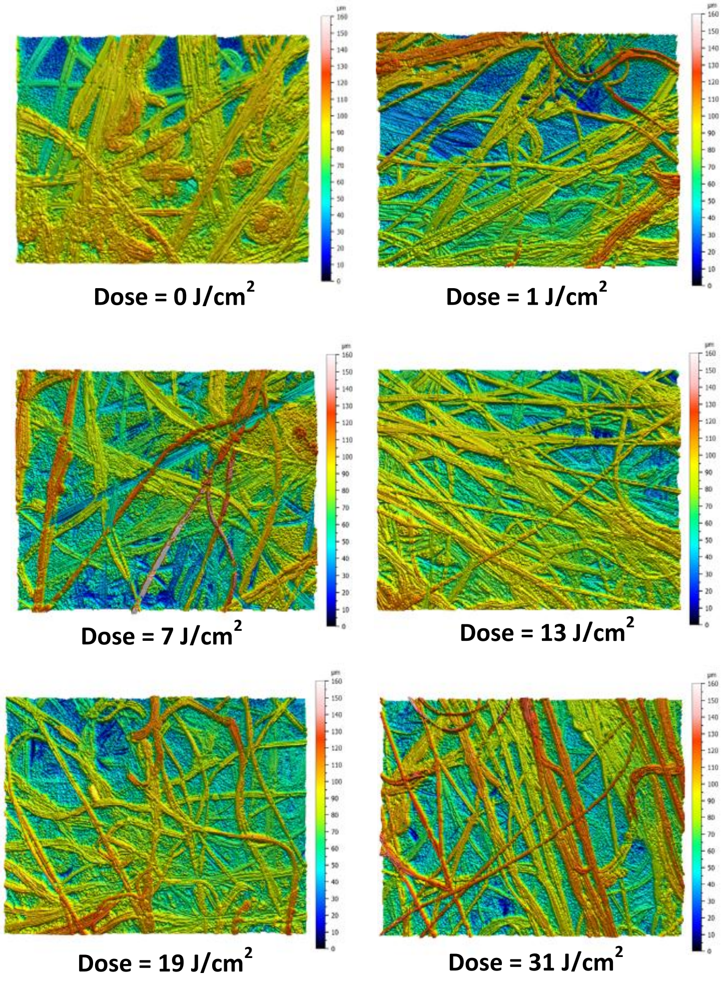

Figure 8. Zeta-20 images of the meltblown layer of the N95 respirator with different UVC doses: (a) 0J/cm$^2$; (b) 1J/cm$^2$; (c) 7J/cm$^2$; (d) 13J/cm$^2$; (e) 19J/cm$^2$; and (f) 31J/cm$^2$. Z scale is 0–160μm for all images. The field of review is 374μm by 281μm

Fiber width analysis was derived from the 3D images by selecting 25 random fibers and measuring the widths. Average fiber width as a function of UVC dose is shown in Figure 9. The data shows that the average fiber width decreases 37% at 1J/cm$^2$ of UVC dose and continues to decrease until a dose of 19J/cm$^2$. During the 0 – 19J/cm$^2$ dosage range, the width data exhibits a strong and well-behaved logarithmic decrease with dosage, with $R^2 = 0.95$ (note that for the 0 to 13J/cm$^2$ dosage range, $R^2 = 0.99$). At a dose of 31J/cm$^2$, the trend saturated and no additional decrease was observed. Fiber width variability was also large for each 25-measurement average, and this variation could be attributed to the waviness and non-uniformity of the meltblown PP fibers.

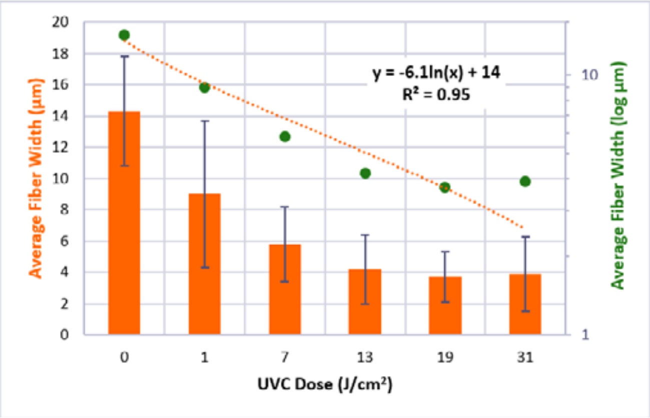

Figure 9. The average fiber width as a function of UVC dose appears to decrease logarithmically with increased dose, up to 19J/cm2. Between 19 and 31J/cm2, the width decrease appears to saturate, so the 31J/cm2 data point was not included in the fit.

## CONCLUSIONS

In this work, the effect of UVC disinfection treatment on the nanomechanical and topographic properties of N95 respirator filtration fibers was investigated using a KLA Nano Indenter G200X with the Continuous Stiffness Measurement (CSM) and survey scanning options, as well as a Zeta-20 optical profiler. When the UVC dosage was raised from 0 to 1J/ cm$^2$, a significant decrease of the nanoindentation hardness and modulus was observed. At higher UVC doses, the average Young's modulus, E, and hardness, H, both continued to decrease with the increase of UVC dose, as measured on individual fibers. These results show that the PP microfiber layer loses its strength when N95 respirators are overexposed to accumulated doses of UVC radiation during the process of decontamination. Loss of fiber strength can lead to small tears or holes in the mask, resulting in lower protection value against Covid-19 exposure. Further, the fiber width also exhibits a logarithmic decrease during UVC exposure, potentially providing openings for the virus to penetrate the mask.

Future work may include characterizing and quantifying the transformation kinetics of fiber-texture agglomeration. This information may help guide comparisons among materials and further improve the stability of N95 mask material constituents and processes.

## ACKNOWLEDGEMENT

The authors acknowledge the contributions of Dr. Peter Tsai, the inventor of the N95 respirator filter, for his suggestions on preparing the manuscript.
